# Förster resonance energy transfer biosensors for fluorescence and time-gated luminescence analysis of rac1 activity

**DOI:** 10.1038/s41598-022-09364-w

**Published:** 2022-03-28

**Authors:** Ha Pham, Mona Hoseini Soflaee, Andrei V. Karginov, Lawrence W. Miller

**Affiliations:** 1 Department of Chemistry, University of Illinois at Chicago, 845 W. Taylor Street, MC 111, Chicago, IL 60607 USA; 2 Department of Pharmacology and The Center for Lung and Vascular Biology, The University of Illinois College of Medicine, Chicago, IL USA

**Keywords:** Microscopy, Chemical tools, Biological techniques, Chemical biology, Chemistry

## Abstract

Genetically encoded, Förster resonance energy transfer (FRET) biosensors enable live-cell optical imaging of signaling molecules. Small conformational changes often limit the dynamic range of biosensors that combine fluorescent proteins (FPs) and sensing domains into a single polypeptide. To address this, we developed FRET and lanthanide-based FRET (LRET) biosensors of Rac1 activation with two key features that enhance sensitivity and dynamic range. For one, alpha helical linker domains separate FRET partners and ensure a large conformational change and FRET increase when activated Rac1 at the biosensor C-terminus interacts with an amino-terminal Rac binding domain. Incorporation of a luminescent Tb(III) complex with long (~ ms) excited state lifetime as a LRET donor enabled time-gated luminescence measurements of Rac1 activity in cell lysates. The LRET dynamic range increased with ER/K linker length up to 1100% and enabled robust detection of Rac1 inhibition in 96-well plates. The ER/K linkers had a less pronounced, but still significant, effect on conventional FRET biosensors (with FP donors and acceptors), and we were able to dynamically image Rac1 activation at cell edges using fluorescence microscopy. The results herein highlight the potential of FRET and LRET biosensors with ER/K linkers for cell-based imaging and screening of protein activities.

## Introduction

Fluorescence-based biosensors play a vital role in cell biology research by making it possible to quantify within living cells the spatiotemporal dynamics of analyte concentrations, signaling protein activities and microenvironmental conditions^1–3^. Many of these biosensors are recombinantly expressed fusion proteins that rely on Förster resonance energy transfer (FRET) to read out changes in protein binding or conformation^4^. FRET is non-radiative, dipole–dipole energy transfer that occurs when luminescent donor and acceptor molecules with compatible absorption and emission spectra reside within ~ 10 nm of one another^5^. Any change in distance or orientation between donors and acceptors that alters FRET efficiency may be dynamically detected as a reduction in donor emission intensity or lifetime or as an increase in acceptor emission intensity coincident with donor excitation, if the acceptor species itself is fluorescent. FRET, then, provides a straightforward way to transduce biochemical changes into optical signals by incorporating FRET pairs and sensing domains into a single genetically encoded polypeptide.

A key performance metric of FRET biosensors is dynamic range, or maximum observable signal difference between active and inactive states. High dynamic range is required to compensate for the generally low signal-to-noise (S/N) seen with intensity-based FRET measurements. Low S/N is a hallmark of FRET microscopy with fluorescent proteins (FPs) because spectral overlap comingles donor-sensitized, acceptor emission signals with donor and directly excited acceptor fluorescence^5^. The deleterious effects of spectral overlap worsen when imaging two freely diffusing fluorophores, especially when there is a large localized difference in either donor or acceptor abundance. Single-chain biosensor designs simplify FRET imaging and data analysis by incorporating sensing domains and fluorophores into a single polypeptide to maintain a 1:1 ratio of donors and acceptors^6^. However, a single-chain affinity biosensor can fold into an off-state, or low-FRET, conformation that positions donor and acceptor in close proximity and yields a high baseline signal. As a consequence, the dynamic range of single-chain biosensors rarely exceeds 50%^7^.

Efforts to increase biosensor dynamic range often require empirical testing of many different geometries that vary in linker length or composition, fluorophore type or domain order. The development of FRET biosensors of Rho family GTPase signaling proteins highlights the different strategies used to optimize biosensor performance^8^. GTPases, including Rho, Rac1 and Cdc42, regulate diverse cellular processes including actin remodeling, adherence junction formation, and establishment of cell polarity^9,10^. Rho GTPases are active when bound to guanosine triphosphate (GTP) and inactive when bound to guanosine diphosphate (GDP). Activity is mediated by various regulator proteins including guanine nucleotide exchange factors (GEFs.), GTPase activating proteins (GAPs) and guanine nucleotide dissociation inhibitors (GDIs)^11–13^. A number of innovative single-chain GTPase biosensors have been reported that incorporate optimized linker designs or circularly permuted (cp) fluorescent proteins (cpFPs) to enhance performance, with some reported examples exhibiting dynamic ranges of 150% or more^14–19^.

In this study, we used Rac1 as a model system to elaborate on our recently reported biosensor design that incorporates rigid, helical linker domains and luminescent terbium (Tb(III)) complexes as energy transfer donors^20,21^. Our objectives were two-fold: (i) to develop LRET-type biosensors with ER/K linkers for multi-well plate TGL assays of Rac1 inhibition; and (ii) to evaluate the effects of ER/K linkers on the dynamic range of FRET-type Rac1 biosensors. So-called ER/K linkers, comprised of alternating repeats of four glutamate residues and four arginines or lysines, form an extended α-helix with hinge-like properties^22^. ER/K linkers enhance dynamic range by separating affinity binding domains and FRET partners in the unbound (low activity) state and thereby reducing baseline FRET signals^23^. In situ labeling of sensor peptides with luminescent Tb(III) complexes enables time-gated luminescence (TGL) detection of luminescence resonance energy transfer (LRET) between Tb(III) donors and GFP acceptors and enables cell-based screening assays of protein–protein interactions characterized by high signal-to-background (S/B) and dynamic range^20^.

We prepared expression vectors with domain order (*N-* to *C-*) of FP, Rac1 binding domain, linker, FP (or Tb(III) complex binding domain) and full-length Rac1. The biosensors featured either flexible linkers or ER/K linkers of varied length and one of three different donor/acceptor pairs: (i) mCerulean/Ypet; (ii) circularly permutated (cp) mTFP1/cp Venus; and iii) Tb(III)/EGFP). We co-expressed each biosensor configuration with upstream regulatory proteins and measured dynamic range of donor-denominated FRET ratios using either fluorometry (for FP-based sensors) or TGL (for LRET sensors). For Tb(III)-based sensors, dynamic range increased with ER/K helix length to a maximum 1100% for sensors with a 30 nm linker. Large signal differences and high apparent Z’ factors were also observed in a 96-well plate LRET assay that measured Rac1 inhibition in cell lysates. FP-based sensor performance in fluorometric assays did not correlate with ER/K linker length. However, ER/K-based, Ypet/mCerulean Rac1 biosensors exhibited significantly enhanced signal changes (up to 125%) relative to equivalent sensors with unstructured linkers. Using FRET microscopy and an engineered cell line that expressed a Ypet/mCerulean sensor with a 20 nm ER/K linker, we observed robust Rac1 activation near protruding edges of stimulated cells. These results, along with our earlier studies^20^, demonstrate that FRET or LRET biosensors with ER/K linkers are a robust platform for engineering sensitive single cell imaging studies or higher-throughput cell-based assays of protein function in live cells.

## Results and discussion

### Biosensor design

In 2000, Kraynov et al. developed FLAIR (fluorescence activation indicator for Rho proteins) to image intracellular Rac1 activity. The FLAIR biosensor relied on FRET to monitor the interaction between a chimera of Rac1 fused to a FP and the p21-binding domain of Pak1 (PBD) labeled with the dye, Alexa546^24^. Pak1 is a downstream effector that binds to the active, GTP-bound state of Rac1. Since the report of FLAIR, steady improvements have been made to Rac1 biosensors and the performance of FRET-based bioimaging more generally^2^. Matsuda and co-workers developed the first single-chain Rac1 biosensor (Raichu-Rac1) that linked Pak1 PBD to Rac1 with FPs at each terminus^15^. In a later study, the same group improved the dynamic range of Raichu-Rac1 three-fold by increasing linker stiffness and using dimerizing FPs^16^. Hodgson and co-workers designed a biosensor with domain order mCerulean, tandem PBDs, mVenus and Rac1 with each domain separated by short linkers (2–14 residues)^18^. Placement of Rac1 at the C-terminus retained the CAAX box that localizes native Rac1 at the plasma membrane and facilitates regulation by RhoGDI^24^. Subsequent optimization with circularly permuted mVenus yielded a two-fold gain in dynamic range, up to 150%^25^. Fritz, et al. engineered a large library (over 70 constructs) of RhoA biosensors that explored linker length, domain order and fluorophore dipole orientation using circularly permuted FPs (cpFPs)^26^. The results of this study ultimately yielded a RhoA sensor with a gain of 150%, representing a three-fold improvement overy the original configuration. Subsequently, they applied a similar methodology to improve Rac1 biosensor response from 40 to ca. 70%, yielding the Rac1-2G biosensor, used as a basis for comparison in the present study^14^.

**Figure 1:**
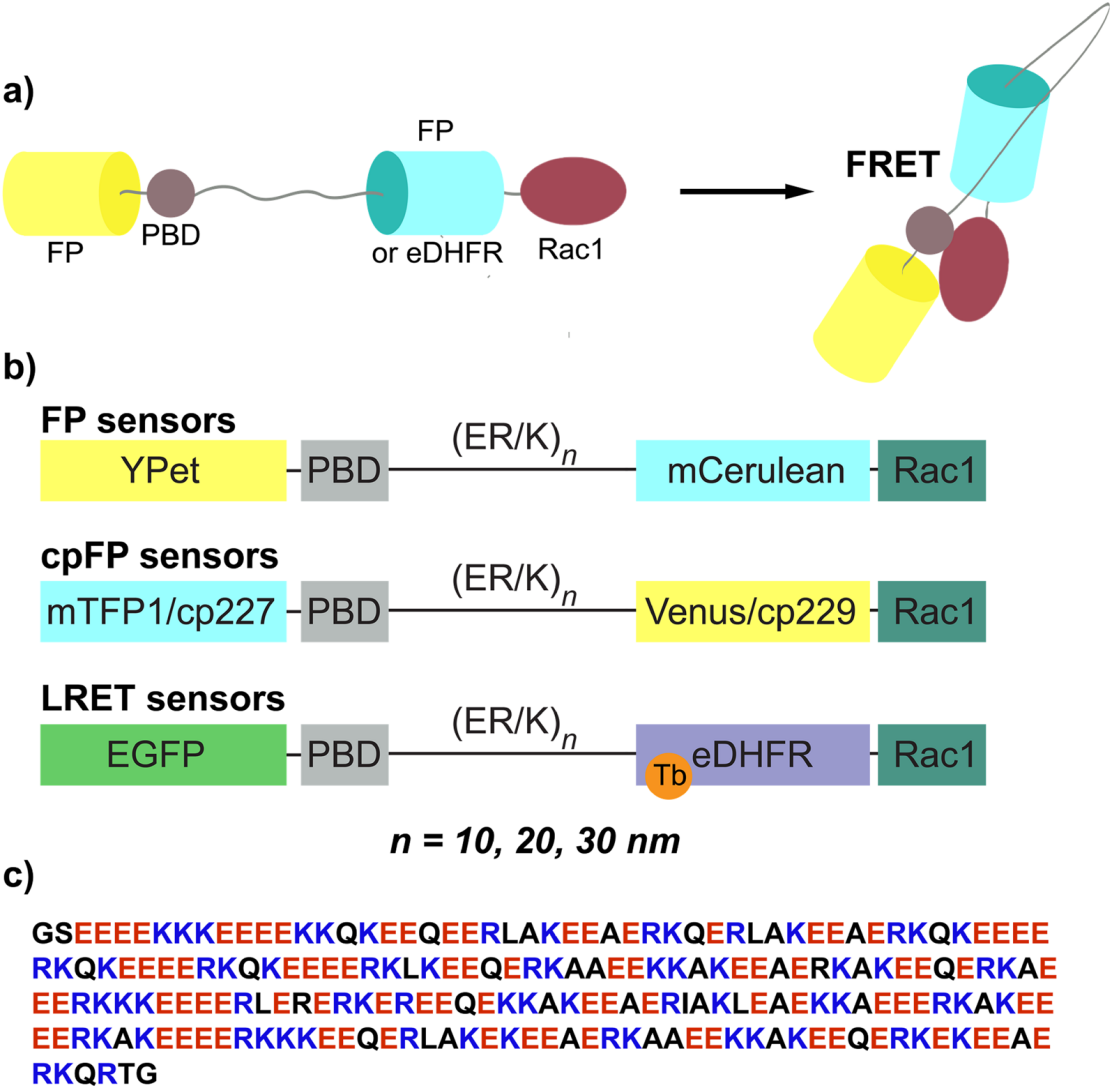
Design of FRET and LRET Rac1 biosensors. (**a**) Schematic representation and (**b**) mammalian expression constructs of single-chain FRET or LRET Rac1 biosensors. A series of nine sensors was constructed in which alpha-helical ER/K linkers of different length (10 nm, 20 nm and 30 nm) were combined with three fluorophore pairs: (i) mCerulean and Ypet; (ii) circularly permutated mutant (cp227) of mTFP1 and circularly permutated mutant (cp229) of Venus; (iii) Tb(III) complex (bound to *E. coli* dihydrofolate reductase, eDHFR) and EGFP. (**c**) Sequence of a 30 nm alpha-helical ER/K linker (207 residues) that includes alternated repeats of approximately four negatively charged glutamates (red) and four negatively charged arginines or lysines (blue). The linker is rigid to keep protein components far apart in OFF state. Stochastic breaks allow protein interaction in the biosensor ON-state and promote a large change in donor-sensitized acceptor emission.

Similar to previously reported designs, our sensors rely on the interaction between the Pak1 PBD (residues 68–150) and the active, GTP-bound form of Rac1 to report changes in Rac1 activity. Each sensor fusion protein incorporated five domains, ordered from *N-* to *C-*terminus as follows: (i) a FRET acceptor FP; (ii) PBD; (iii) an ER/K linker sequence (length of 10, 20 or 30 nm); (iv) either a FRET donor FP or *Escherichia coli* dihydrofolate reductase (eDHFR) that binds to a trimethoprim (TMP)-Tb(III) complex conjugate; and (v) full-length Rac1 (Fig. 1a). We prepared biosensors with three different FRET/LRET pairs that included mCerulean/Ypet, mTFP1(cp227)/Venus(cp229), and eDHFR(Tb)/EGFP (nine total sensors, Fig. 1b). The biosensors that included mTFP1(cp227)/Venus(cp229) were based on the Rac1-2G biosensor^14^, Sensors with mCerulean and Ypet were based on a heretofore (to our knowledge) unpublished construct that incorporated a long (78 residue) flexible middle linker (see Experimental Methods for details on all constructs).

We previously showed that biosensors which incorporate Tb(III) complexes as LRET donors, EGFP as LRET acceptors and ER/K linkers can be used to craft cell-based TGL assays of protein–protein interactions that exhibit exceptional sensitivity and dynamic range^20^. Inclusion of an eDHFR domain permits self-assembly with TMP-Tb(III) complex conjugates inside live cells or in cell lysates. TGL detection of LRET offers high signal-to-background ratios that derive from the ms-scale emission decay times of Tb(III) luminescence and Tb(III)-to-GFP sensitized emission. TGL plate readers or microscopes implement a brief delay (~ µs) between pulsed, near-UV excitation and detection that nearly eliminates non-specific fluorescence (with ~ ns decay times) from cells, library compounds or directly excited acceptors.

The ER/K linker forms an extended alpha helix that behaves as a worm-like chain with a persistence length (*L*_*p*_) of about 15 nm^22^. Accordingly, the ER/K helix acts as a rigid rod at lengths ≤ *L*_*p*_ that serves to keep apart FRET partners and binding domains to minimize baseline energy transfer when the sensor is in the unbound or off state. Sivaramakrishnan and Spudich showed that ER/K linkers limit *k*_*close*_ of terminal affinity domains in FRET biosensors but do not affect *k*_*open*_, and they presumed that random breaks imbue hinge-like properties to the helix that permit close approach of ends and interaction of binding partners. Consequently, the proportion of biosensors in the closed and open conformations depends solely on the ER/K linker length and the inherent affinity of the binding partners^23^. We anticipated that ER/K linkers would increase dynamic range in a length-dependent manner and prepared of FRET and LRET biosensors with linkers of approximately 10 nm (73 residues), 20 nm (131 residues), or 30 nm (207 residues; sequence, Fig. 1c). By comparison, the parent biosensors with mCerulean/YPet and mTFP1(cp227)/ Venus(cp229) as FRET partners had flexible linkers of length 78 and 69 residues, respectively.

### Biosensor characterization

**Figure 2:**
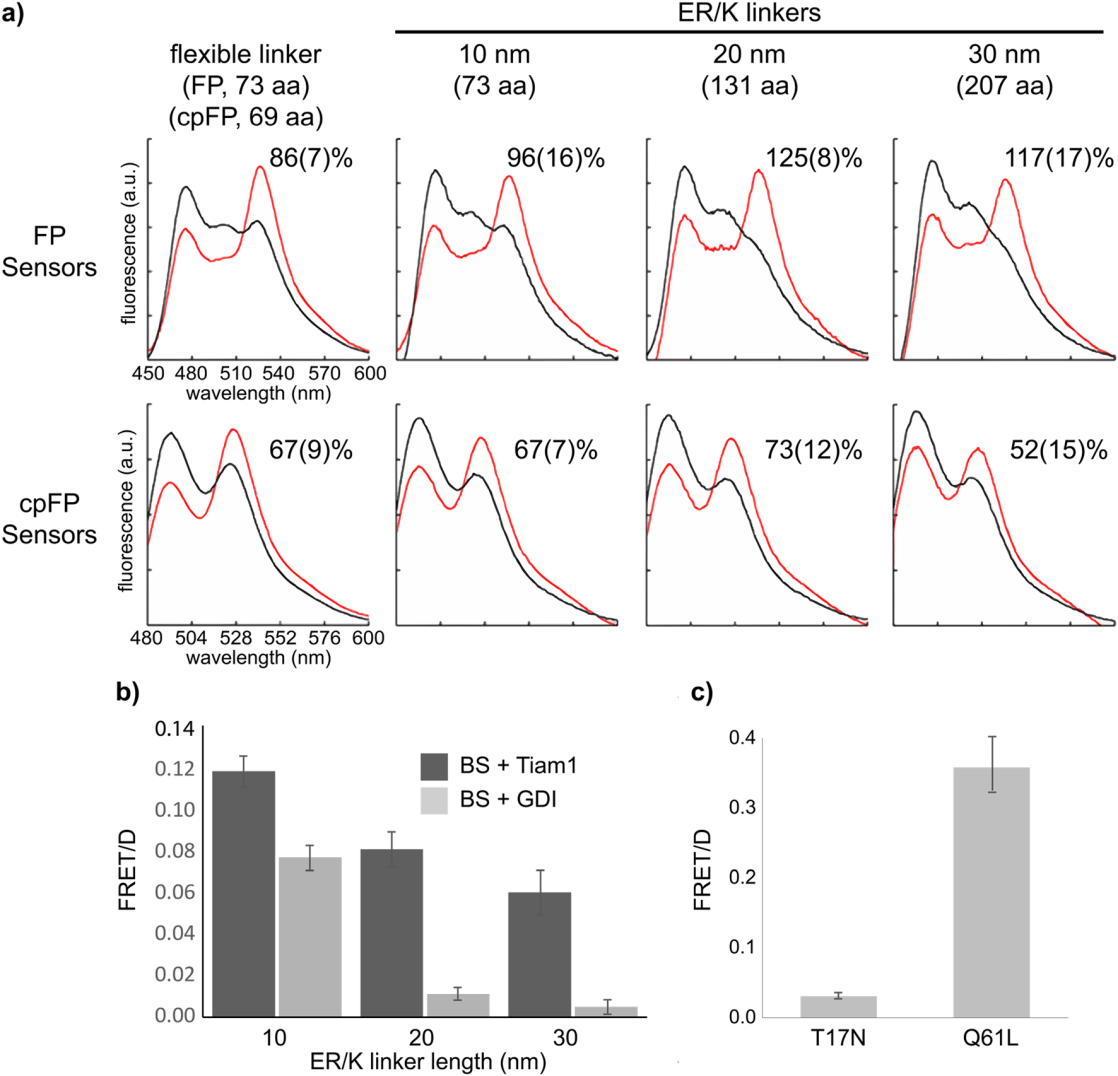
Evaluation of Rac1 biosensors by fluorometry and TGL luminescence. Rac1 biosensors were evaluated by co-expressing each sensor with Tiam1 (On state, red trace in (**a**) or black bar in (**b**) or RhoGDI (Off state, black trace in (**a**) or gray bar in (**b**) in 293 T cells. (**a**) Cells co-expressing FP or cpFP sensors and the regulators were scanned for emission profiles in cell suspension using a cuvette-based fluorometer. ΔR/R_i_ values representing differences in FRET efficiency between the On and Off states are shown for the indicated biosensor. (**b**) Cells co-expressing LRET biosensors and the regulators were grown in a 96-well plate. Following overnight incubation, cells were treated with lysis buffer containing TMP-TTHA-cs124 (25 nM). The time-gated emission (gate delay, 0.2 ms) at 520 nm (Tb-to-GFP LRET) and 615 nm (Tb only) were measured using a time-resolved fluorescence plate reader. Substantially larger 520 nm/615 nm (FRET/D) ratios were observed in the positive control wells (Tiam1) relative to those seen in the negative controls (RhoGDI). (**c**) 293 T cells expressing inactive (Rac1 T17N) or active (Rac1 Q61L) mutants of the LRET biosensor with 30 nm ER/K linker were grown in a 96-well plate and underwent the same lysis-buffer treatment and plate-reader measurement as in (**b**). Fluorescence spectra in (**a**) are representative of cells transiently expressing biosensors with transfection efficiency > 70%. Dynamic range values represented as mean(sem) (n ≥ 3 transfections). Error bars in (**b**), (**c**), sem (n ≥ 3 transfections/plates; 16 wells/plate for each condition).

We evaluated dynamic range by co-expressing biosensors along with Rac1 upstream regulators and using a fluorometer (for FP or cpFP biosensors) or a TGL plate reader (for LRET biosensors) to read out emission signals. We transiently co-transfected HEK293T cells with plasmid DNA that encoded a biosensor and either Tiam1 or GDI (4:1 regulator/biosensor DNA ratio) to maintain sensors in the Rac1-active or inactive states, respectively. Following overnight expression, we obtained scanning fluorescence emission spectra of suspended cells (Fig. 2a). For plate reader assay, 293 T cells co-expressing LRET biosensors and Tiam1 or GDI were seeded in a 96 well plate. A lysis solution containing a TMP-Tb(III) conjugate (TMP-TTHA-cs124) was added to the cells approximately ten min before TGL measurement of Tb(III) and Tb(III)-to-GFP emission intensity (see Experimental Methods). We calculated biosensor dynamic range as (R_a_-R_i_)/R_i_, or ΔR/R_i,_ where R_a_ and R_i_ were the sensitized acceptor (FRET)-to-donor emission ratios obtained from spectra of cells expressing activated (with TIAM1) and inactivated (with GDI) biosensors, respectively.

Based on our prior studies^20^, we expected to see a large difference in donor-denominated FRET or LRET ratios between GDI and TIAM1 cells that increased with ER/K linker length. However, the incorporation of ER/K helical linkers had little effect on the performance of FP (mCerulean/YPet) or cpFP (mTFP1cp227/mVenuscp229) biosensors. The FRET ratios measured in mCerulean/YPet biosensor cells that co-expressed TIAM1 (On-state, Rac1 active) exceeded those measured in GDI co-expressing cells (Off-state, Rac1-inactive) by a range of 86% to 125% (Fig. 2a). Only the mCerulean/YPet biosensor with a 20 nm ER/K linker showed a significantly (*P* < 0.01) improved dynamic range in comparison to the same configuration that incorporated an unstructured linker in a statistically significant manner (125 ± 8% vs. 86 ± 7%). The dynamic range value that we observed for Rac1-2G (67%) was nearly same as the literature value (69%)^14^, and values for cpFP anlogs with ER/K linkers ranged from 52 to 73% with no significant correlation to linker length (Fig. 2a).

By contrast, the observed dynamic ranges of Tb(III)-based biosensors strongly depended on ER/K linker length. LRET sensors with 10 nm, 20 nm, or 30 nm linkers exhibited maximum differences in ΔR/R_i_ values of 54(± 16)%, 630(± 30)% and 1100(± 50)%, respectively (Fig. 2b). To further validate this result, we performed the same plate reader assay on cells that expressed active (Rac1 Q61L) or inactive (Rac1 T17N) mutants of the LRET biosensor with 30 nm linker. The difference in LRET/donor emission ratio between active and inactive mutants (1050 ± 100%) was similar to that seen in the Tiam1/GDI assay (Fig. 2c).

The unique properties of the Tb(III) complex as a donor can partly explain the robust dynamic range of LRET sensors. In essence, the narrow emission bands of the Tb complex minimize bleedthrough, and the TGL detection of long-lived donor and FRET signals eliminates background fluorescence. Therefore, even without purifying proteins after lysing cells, our plate reader assay still gave distinct signals between two states of the biosensors. Moreover, the low protein concentration in a plate well (< 10 nM) is typically below the detection limit of FP or cpFP biosensors, highlighting another advantage of LRET-based sensors for TGL analysis.

The characterization data illustrate that longer ER/K linkers significantly decreased background, or Off-state LRET while less dramatically reducing On-state LRET (Fig. 2b). The reduction in Off-state LRET is related to the extended conformation of the ER/K helix. Behaving as an ideal worm-like chain (WLC) with a persistence length (*L*_*p*_) of 15 nm, the mean end-to-end distances for ER/K α-helices with lengths of 10, 20, and 30 nm are 9, 16, and 23 nm, respectively^23^. Appreciable reduction in baseline LRET is still observable at distances ≥ 20 nm because LRET Förster distances-the inter-chromophore distance at which energy transfer efficiency is 50%-are typically longer (5–10 nm) than those seen with conventional FRET (3–7 nm)^27^. The reason that maximal (On-state) LRET signals also decrease is that the effective concentration of binding partners located each end of the sensor is reduced in a linker length-dependent manner. It was estimated that each 10 nm increase in ER/K linker length reduces effective concentration by an order of magnitude; from 10 µM at 10 nm to 100 nM at 30 µM^23^. Consequently, as linker length increases, the maximum fraction of the sensor in the closed state decreases as well.

While the ER/K linker lengths tested here substantially impacted the performance of LRET Rac1 biosensors, they had minimal effect on that of FP- or cpFP-based biosensors. We observed a 40% increase in the dynamic range of the mCerulean/Ypet sensor with a 20 nm (131 residue) linker relative to its counterpart with a flexible (78 residue) linker. However, none of the other constructs showed significant changes. It may be that ER/K linkers of 10 to 30 nm are too long to substantially improve the dynamic range of single-chain FRET sensors. For example, with a Förster distance of ~ 5 nm, a distance between FPs of 10 nm would be expected to reduce FRET efficiency more than 98%. Further separation, with longer ER/K linkers, would then reduce effective concentrations and maximal on-state FRET. Moreover, the mCerulean/Ypet and mTFP1(cp227)/mVenus(cp229) biosensors that we tested for comparison had relatively long linkers of 78 and 69 residues, respectively. Long, flexible linkers also reduce off-state FRET efficiency, although not in proportion to linker length^16,28,29^. For example, constructs of ECFP and YFP joined together by (GGSGGS)_n_ linkers with lengths ranging from 23 to 71 residues had observed FRET efficiencies of 0.71 to 0.43 and mean inter-chromophore distances of 3.9 to 5.3 nm^29^. To our knowledge, the effects of shorter ER/K linker lengths (< 10 nm, ~ 70 residues) on FP-FRET dynamic range have not yet been explored and bear further study. Another factor to consider in future studies is the extent to which rigid ER/K linkers restrict fluorophore mobility and unfavorably affect the relative dipole orientation orientations of FP donors and acceptors. In contrast to FPs, lanthanide complexes emit at multiple dipole orientations, and LRET efficiency is independent of donor and acceptor orientation^27^.

### Biosensors in live cells

**Figure 3:**
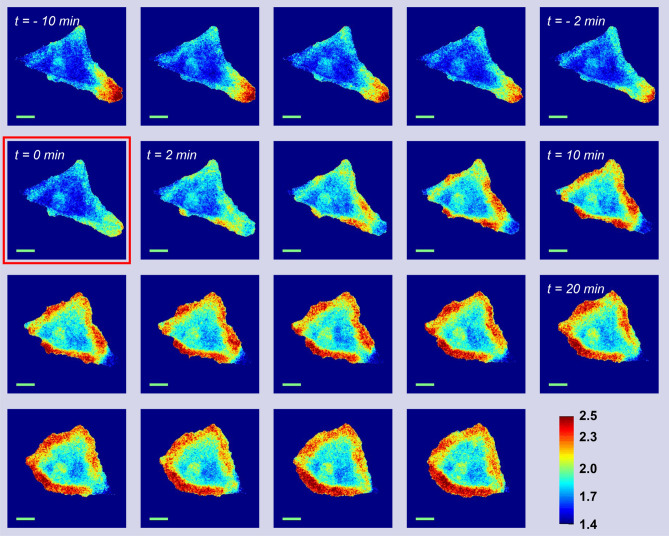
Live-cell imaging of Rac1 biosensors. Representative image series of a HeLa cell co-expressing the FP Rac1 sensor with the 20 nm ER/K linker (transient transfection) and RapR-Src constructs (stable transfection) was imaged at two-minute intervals. The first image obtained following rapamycin addition (500 nM) is indicated by the square (at *t* = 0). The montage shows FRET/mCerulean ratio images with warmer colors reflecting higher localized Rac1 activity.

We next evaluated the functionality of a FP-based sensor with an ER/K linker for FRET microscopic imaging of Rac1 in HeLa cells. In imaging studies, Rac1 activity in sensor-expressing cells has been induced with growth factors (EGF, PDGF) or other extracellular stimuli^24^. However, this approach triggers a multitude of parallel signaling pathways and often a brief, transient activation of Rac1 which significantly complicates the analysis of sensor activity. To simplify our imaging characterization experiments, we applied a recently developed protein engineering method that employed a rapamycin-regulated allosteric switch to regulate tyrosine kinase c-Src (Src) activation^30^. In this way, activation of Rac1 could be controlled indirectly by rapamycin-induced Src (RapR-Src) activation. For live-cell imaging, cells co-expressing the mCerulean/YPet Rac1 biosensor with 20 nm ER/K linker and RapR-Src constructs were seeded on a fibronectin-coated chamber slide. Cells were imaged every 2 min for 20 min before and 40 min after adding rapamycin. We observed robust Rac1 activation near protruding edges of stimulated cells following rapamycin addition. Cells started vigorous spreading and protrusion a few minutes after stimulation. Increased sensor activity was consistently observed wherever protrusions were extending (Fig. 3).

### Detection of Rac1 inhibition in multi-well plates

Increasing evidence supports the involvement of Rac1 signaling alterations in cancers^31,32^. Because mutations in Rac1 proteins are sporadic, the mechanism of Rac1 in cancer likely occurs through its overexpression or hyperactivity^33^. In fact, overexpression of Rac1 and its hyper-activation caused by overexpression of Rac1 specific GEFs such as Tiam1 and Vav1 have been observed in various tumor types, including pancreatic cancer^34,35^. Accordingly, the inhibition of Rac1 is speculated to have an antiproliferative effect on cancer cells^32^. Several small molecules have been reported as Rac1 inhibitors^36–40^. NSC23766 and EHT 1864 were among the first developed Rac1 inhibitors with the capability to discern from other Rho family GTPases, such as Cdc42 or RhoA^39,40^. The former impedes Rac1 activation by occupying the binding location of two Rac1 GEFs: Trio and Tiam1^39^, while the latter keeps Rac1 in an inactive state and prevents its binding to downstream effector^40^, although the selectivity of each inhibitor has been called into question^41,42^. Small molecule inhibitors that target Rac1 have been identified through structure-based virtual high throughput screening^36,38^, followed by pull-down assays to examine hits^43,44^. Bead-based flow cytometry assays and a high-content imaging screen of Rac1 activity have also been reported^45,46^. We showed that single-chain LRET biosensors could be adapted for medium- and high-throughput assays of protein interactions in cell lysates and intact cells using widely available TGL plate readers^20^.

**Figure 4:**
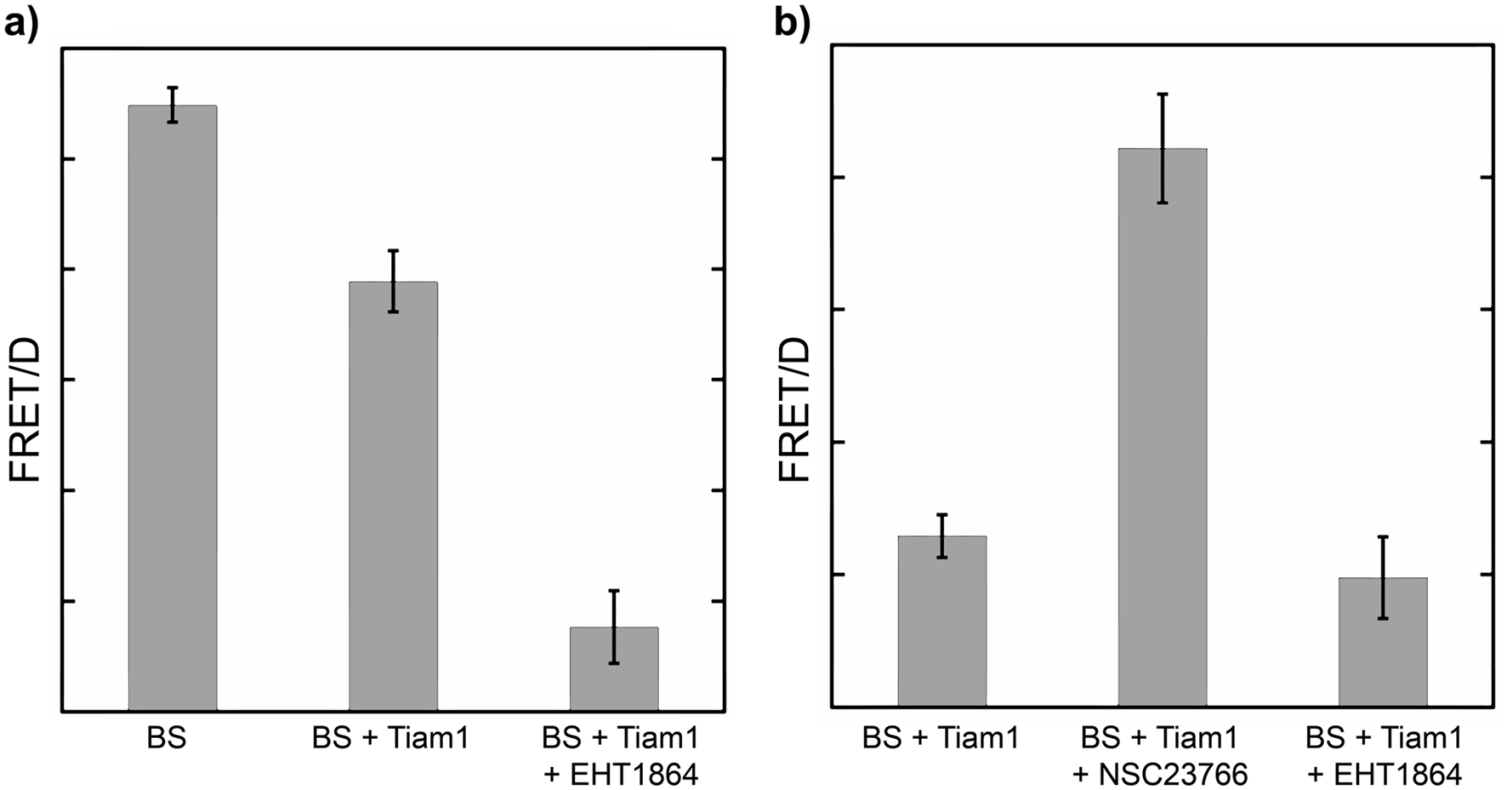
LRET robustly detects chemical inhibition of Rac1 activitation in 96-well plates. (**a**) 293 T cells expressing a LRET Rac1 biosensor (30 nm ER/K linker) alone (BS) or with Tiam1 (BS + Tiam1) were grown overnight in 96-well plates, then incubated with. Time-gated measurements were obtained following 4 h incubation with EHT 1864 (50 µM, 0.5% DMSO) and addition of a lysis buffer containing TMP-TTHA-cs124. (**b**) 293 T cells co-expressing the sensor and Tiam1 were grown in 96-well overnight with or without inhibitors in the media. Time-gated measurements were taken after adding the lysis buffer as in (**a**). Bar graphs depict mean FRET/donor (FRET/D) ratios measured for each condition. Error bars, sem (n ≥ 3 transfections/plates; 16 wells/plate for each condition).

We sought to assess the potential of our LRET Rac1 biosensor for the detection and quantification of Rac1 inhibition in a multi-well plate format by measuring the effects of NSC23766 and EHT 1864 on biosensor activity. We co-transfected 293 T cells in 96-well plates with LRET biosensor (30 nm ER/K linker) and Tiam1 DNA. After 48 h, the cells were incubated with growth media containing inhibitor (positive controls, NSC23766 or EHT 1864 50 µM) or vehicle (negative control, 0.5% DMSO) for four hours or overnight (See Experimental Methods for sample handling protocols). Following addition of lysis buffer containing Tb(III) complex (TMP-Lumi4-Tb, 25 nM), we measured time-gated Tb(III)-to-GFP and Tb(III) emission signals at 520 nm and 615 nm, respectively. After four-hour incubation with EHT 1864 (50 µM), we observed > 70% reduction in the LRET/Tb (520 nm/615 nm) emission to approximately the same level as the biosensor without Tiam1 activation (Fig. 4a). Compared to EHT1864, overnight incubation with NSC23766 only partially inhibited Rac1 (Fig. 4b). In order to assess statistical robustness, we calculated apparent Z’ factors (single plate/transfection condition, n = 16 wells). For inhibition assays with EHT 1864, Z’ ranged from 0.5 to 0.7, and with NSC23766, Z’ ranged from 0.05 to 0.2 (n ≥ 3 experiments for each condition, see Experimental Methods for data handling and statistics).

## Conclusion

LRET biosensors’ extraordinary dynamic range stems from time-gated detection of LRET that eliminates non-specific fluorescent background and from the incorporation of ER/K linkers that maintains the donor and acceptor far apart in the open sensor configuration. These features enable robust detection of Rac1 activation or inhibition in cell lysates in 96-well plates and support medium or high throughput detection of small molecules inhibitor for Rac1 or any other proteins, especially proteins that are difficult to purify. Moreover, Tb(III) can sensitize differently colored acceptors, offering the potential for multiplexed imaging or analysis^47^.

The extent to which rigid, helical linkers benefit conventional FRET biosensors is less clear, as we observed significant enhancement relative to unstructured linkers in only one case. Nevertheless, the ability to precisely control chromophore separation in FRET biosensors with a rigid, yet hinge-like linker remains intriguing. This study and others published thus far have only explored ER/K linkers of lengths ranging from 10 to 30 nm (ca. 70 to 200 residues)^48^. Further exploration of ER/K linkers at length scales < 10 nm would more fully elaborate the potential benefits of these structures for biosensor design.

## Experimental methods

### Materials

293 T cells (CRL-3216), HeLa cells (CCL-2), and MEF cells (SCRC-1040) were from ATCC. Dulbecco’s modified eagle medium with 4.5 g/L glucose (DMEM, 10-013CV), Dulbecco’s phosphate buffered saline (DPBS, 21–030 and 21–031), and 0.05% trypsin/2.21 mM EDTA (Corning, 25–053-Cl) were purchased from Corning cellgro®. DMEM (without phenol red, 21,063), HEPES (15,630–080) and Lipofectamine 2000 (11,668–027) were purchased from Invitrogen™. FBS (S11150) was purchased from Atlanta Biologicals. Hygromycin (sc-29067) was purchased from Santa Cruz Biotechnology. BSA (700-107P) was purchased from Gemini Bio-products. Rapamycin (553,211-500UG) and Fibronectin bovine plasma (F1141) were purchased from Millipore. NADPH (N0411) and doxycycline (D9891) were purchased from Sigma. DMSO (D128-500) was purchased from Fisher Chemical. Patent V blue sodium salt (21,605) was purchased from Fluka. In-Fusion HD cloning kit (638,909) was purchased from Takara. All enzymes and buffers used in cloning were purchased from New England Biolabs. pUSE-RapR-Src-as2-mCherry-myc and pUSE- ipep-FRB^*^ plasmids were a gift from Andrei Karginov (University of Illinois at Chicago)^30^. pTriEX-Ypet-PBD-mCerulean-Rac1 (Rac1-Flare.sc) was a gift from Klaus Hahn (University of Noth Carolina at Chapel Hill). pTriEx4-Rac1-2G^14^ was a gift from Olivier Pertz (Addgene plasmid # 66,110; http://n2t.net/addgene:66110; RRID: Addgene_66110). The plasmids, pPBH-TREtight-FRB-eDHFR-(ER/K)_*n*_-EGFP-FKBP12 (*n* = 10, 20, 30 nm)^20^, were used as source DNA for ER/K linkers of the indicated lengths. The amino acid sequence of the 30 nm ER/K linker (207 residues) is indicated in Fig. 1, and the 20 nm ER/K linker comprises the first 131 residues of the 30 nm sequence. The sequence of the 10 nm linker (73 residues) is EEEEKKKQQEEEAERLRRIQEEMEKERKRREEDEKRRRKEEEERRMKLEMEAKRKQEEEERKKREDDEKRKKK. Heterodimers of trimethoprim linked to luminescent Tb(III) complexes (TMP-cs124-TTHA)^49,50^ were prepared as previously reported.

### Cell culture

HeLa cells were maintained in DMEM (1.0 g/L glucose) supplemented with 10% FBS, 1X MEM non-essential amino acids and 15 mM HEPES at 37 °C and 5% CO2. The cells were passaged with 0.25% trypsin/2.21 mM EDTA. 293 T cells were maintained in DMEM (4.5 g/L glucose) supplemented with 10% heat-inactivated FBS and 2 mM L-glutamine at 37 °C and 5% CO2. The cells were passaged with 0.05% trypsin/2.21 mM EDTA. NIH 3T3 cells were maintained in DMEM (4.5 g/L glucose) supplemented with 10% FBS at 37 °C and 5% CO2. The cells were passaged with 0.05% trypsin/2.21 mM EDTA.

### Plasmids

All DNA constructs were sequenced by the UIC Research Resources Center (RRC).

*pTriEX-Ypet-PBD-(ER/K)*_*n*_*-mCerulean-Rac1 (n* = *10 nm, 20 nm, or 30 nm)* was prepared by subcloning from pTriEX-Ypet-PBD-mCerulean-Rac1. HindIII to NotI fragments encoding 10 nm, 20 nm, or 30 nm ER/K linker were amplified using the following primer pairs respectively: 5’-ACT GAA GCT TCA GGA AGC GGA GAA GAG GAA GAG A-3’ and 5’-TGA TGC GGC CGC CAG AGC CCT TCT TCT TGC G-3’; 5’-ACT GAA GCT TCC GGA GGA TCC GAA GAG GAG GA-3’ and 5’-TAA TGC GGC CGC CAG AGC CAC CGG TCT CT-3’; 5’-AGC AAA GCT TCT GGA TCC GAA GAG GAG GAG A-3’ and 5’-CTT AGC GGC CGC CAC CGG TTC TCT GTT TTC GC-3’. The linker fragment was then inserted between the HindIII site and the NotI site in the source vector.

*pTriEX4-mTFP1/cp227-PBD-(ER/K)*_*n*_*-Venus/cp229-Rac1 (n* = *10 nm, 20 nm, or 30 nm)* was subcloned from pTriEX4-Rac1-2G. XmaI to NotI fragments encoding 10 nm, 20 nm or 30 nm ER/K linker were amplified using the following primer pairs respectively: 5’-TAA TCC CGG GGG AAG CGG AGA AGA GGA AG-3’ and 5’-TAA TGC GGC CGC GCC AGA GCC CTT CTT CTT GC-3’; 5’-TAA TCC CGG GGG AGG ATC CGA AGA GGA GGA GAA-3’ and 5’-TAA TGC GGC CGC AGA GCC ACC GGT CTC TTC-3’; 5’-CGT ACC CGG GGG AGG ATC CGA AGA GGA GGA-3’ and 5’-CTT AGC GGC CGC ACC GGT TCT CTG TTT TCG-3’. Those fragments were then inserted between the XmaI site and the NotI site in the source vector.

*pTriEX4-EGFP-PBD-(ER/K)*_*n*_*-eDHFR-Rac1 (n* = *10 nm, 20 nm, or 30 nm)* was subcloned by replacing mTFP1/cp227 and Venus/cp229 in pTriEX4-mTFP1/cp227-PBD–(ER/K)_n_-Venus/cp229-Rac1 with EGFP and eDHFR. The EGFP fragment was subcloned between NcoI and BspEI sites using the primer pair: 5’-TCG CCA CCA TGG TGA GCA AG-3’ and 5’-TTC GAA GCT TGA GCT CGA GAT CTG-3’. The eDHFR fragment was subcloned between NotI and KpnI sites using the primer pair: 5’-ATG CAG CGG CCG CCA TGA TCA GTC TGA TTG CGG CGT TA-3’ and 5’-ATC GAG GTA CCA GAC CGC CGC TCC AGA ATC TCA-3’.

### Doxycycline-inducible constructs

To generate the dual-promoter plasmid: *pPBH-TRE*_*tight*_-RapR-Src-as2-mCherry-myc and pCMV-ipep-FRB^*^, the gene encoding RapR-Src-as2-mCherry-myc and (CMV Promoter)- ipep-FRB^*^-(bGH Poly(A) Signal Sequence) from pUSE-RapR-Src-as2-mCherry-myc and pUSE- ipep-FRB^*^, respectively, were subcloned to a PiggyBac vector. The fragment encoding RapR-Src-as2-mCherry-myc was amplified by PCR from pUSE-RapR-Src-as2-mCherry-myc by using the primer 5ʹ-ATG CAG CTA GCA TCA TGG GCA GCA ACA AGA GC-3ʹ (BmtI coding strand) and 5ʹ-ATG CAT CTA GAA TCA CCA GTT TCT TCC GGA CTT GTA C-3ʹ (XbaI non-coding strand). This fragment was inserted at the BmtI and XbaI site in the PiggyBac vector. The fragment encoding ipep-FRB^*^ was amplified by PCR from pUSE-ipep-FRB^*^ by using the primer 5ʹ -GCG GCG CCC TGC CCG TCC CAC CAG GTG AGT TCC GCG TTA CAT AAC TTA CGG-3ʹ (SexAI, coding strand), and 5ʹ-GGC CGG TTA CCG CCT GTT GAC CTG GTC GCG TTA AGA TAC ATT GAT GAG TTT GGA C-3ʹ (SexAI, non-coding strand). This fragment was inserted at the SexAI site in pPBH-TREtight- RapR-Src-as2-mCherry-myc using In-Fusion® Cloning Kit.

### Fluorometry assays

pTriEX-Ypet-PBD-mCerulean-Rac1, pTri-EX-Rac1-2G, pTriEX-Ypet-PBD-(ER/K)_n_-mCerulean-Rac1 or pTriEX4-mTFP1/cp227-PBD-(ER/K)_n_-Venus/cp229-Rac1 (n = 10 nm, 20 nm, or 30 nm) biosensor constructs were transiently co-transfected with upstream regulators (Tiam1 or GDI) into 293 T cells plated 1 × 10^5^ per well overnight in 6-well plates, using Lipofectamine 2000 (Invitrogen) according to the manufacture’s protocols. The total amount of DNA was 750 ng per well (150 ng of the biosensor and 600 ng of the regulator). At 48 h after transfection, cells were detached with brief trypsin treatment and were resuspended in 500 μL of ice-cold PBS. Cell suspensions were transferred into a quartz cuvette. Their fluorescent spectrum was then recorded by a fluorometer. The FP and cpFP biosensors were excited at 433 nm and 460 nm respectively, and their emission spectra were recorded from 450 to 600 nm and 480 to 600 nm respectively. Each spectrum was background-subtracted using the spectrum of a suspension of non-transfected cells. The background-subtracted spectra were each normalized to their spectrum integral in order to account for differences in cell number and transfection efficiency, and emission ratios were calculated at the peak wavelengths.

Each series of four sensors (cpFP or FP) was evaluated together at least three separate times in a batch process (eight co-transfections) under the same conditions (cell passage, number of cells, amount of DNA and transfection reagents). Reported dynamic range values represent the mean (± sem, n ≥ 3) percent emission ratio change observed between sensors co-transfected with Tiam1 (FRET-On) and GDI (FRET-Off).

### Multi-well plate assays with permeabilized mammalian cells

LRET biosensor constructs were evaluated by measuring TGL luminescence using a PerkinElmer Victor 3 V multilabel counter with the settings of delay time, 0.2 ms; window time (counting time), 0.7 ms; cycling time, 1.2 ms; excitation wavelength, 340 nm (60 nm bandpass); and emission wavelengths, 520 nm (20 nm bandpass, (Tb-to GFP-LRET) and 615 nm (17 nm bandpass, Tb(III) luminescence). Biosensor constructs (pTriEX4-EGFP-PBD-(ER/K)_n_-eDHFR-Rac1; n = 10 nm, 20 nm, or 30 nm) were co-transfected with upstream regulators (Tiam1 or GDI) into 293 T cells using Lipofectamine 2000. One day prior to transfection, cells were seeded at 9000 cells per well (n = 16 wells for each transfection condition) in a poly-L-lysine coated 96-well plate. The amount of DNA per well was 4.25 ng of the biosensor and 17 ng of the regulator. After 48 h, growth media in the wells were discarded carefully and 70 uL of lysis buffer (5 μM NADPH, 0.1% BSA, 0.1% Triton X-100 in DPBS) with Tb(III) complex (TMP-cs124-TTHA, 25 nM) was added into the wells. The plate was kept at room temperature in dark for 10 min Prior to the first measurement. Background control wells (n = 8 wells) received the same transfection media but no biosensor construct and the same lysis buffer with Tb(III) complex.

The Tb(III) emission (615 nm) and Tb(III)-to-GFP sensitized emission (LRET, 520 nm) signals were measured for each plate. The LRET signal for blank cells that contained buffers, Tb(III) reagents and non-expressing cells was averaged. The mean LRET background was subtracted from the measured LRET value of each individual sample well to obtain background-corrected LRET signals. The background-corrected LRET signals were divided by the corresponding Tb(III) signals to obtain LRET/Tb ratios. Means and standard errors were calculated from the sets of all calculated ratios (n ≥ 3 experiments; 16 wells per plate/experiment).

### Inhibition assay with permeabilized mammalian cells

pTriEX4-EGFP-PBD-(ER/K)_30_-eDHFR-Rac1 biosensor construct were co-transfected with upstream regulator (Tiam1) into 293 T cells using Lipofectamine 2000. One day prior to transfection, cells were seeded at 9000 cells per well in a poly-L-lysine coated 96-well plate. The amount of DNA per well was 4.25 ng of the biosensor and 17 ng of the regulator. After 24 h, cells were incubated with 100 uL of growth media containing NSC23766 or EHT1864 inhibitor (final concentration 50 uM, 0.5% DMSO) for 4 h or overnight. Negative control wells received growth media without the inhibitor but with 0.5% DMSO. Next, growth media in the wells were discarded carefully and 70 uL of lysis buffer (5 μM NADPH, 0.1% BSA, 0.1% Triton X-100 in DPBS) with TMP-cs124-TTHA (25 nM) was added into the wells. The plates were kept at room temperature in dark for 10 min prior to the first measurement. Background control wells received the same transfection media but no biosensor construct and were treated the same as sample wells for the rest of the experiment. Each transfection condition was tested on at least three different days. Means and standard errors were calculated from the sets of calculated ratios for all data (n ≥ 3 plates; 16 wells/plate). Estimated Z’-factors were calculated from a single plate/experiment, and the range of calculated values is presented in the text.

### Stable expression of biosensor plasmids

Rac1 biosensor constructs in PiggyBac vector system were transfected into MEF cells using Lipofectamine 2000. Hygromycin selection was applied to generate the stable cell lines. The cells were then FACS sorted to obtain a population of uniform but medium biosensor expression levels. For imaging experiments, cells were induced by adding 100 ng/mL doxycycline and appropriate biosensor expression levels were achieved at 24 h following the induction.

### Cell imaging of FP Rac1 biosensor with 20 nm ER/K linker

HeLa cells stably expressing RapR-Src-as2-mcherry and ipep-FRB were plated on a fibronectin-coated 25 mm–diameter glass coverslip by placing the coverslip inside a well of a 6-well plate and adding 35,000 cells/well. Cells were incubated for 2–4 h. Before imaging, coverslips were placed into an Attufluor Cell Chamber (Invitrogen, catalog no. A78-16) with Ham’s f12 K medium (no red) containing 10 mM HEPES, DL lactate, and Oxy Fluor, supplemented with 1% (vol/vol) FBS. Live-cell imaging was done using an Olympus IX-83 microscope controlled by Metamorph software and equipped with a heated stage (Warner Instruments), Olympus UPlanSAPO 40 × (oil, N.A. 1.25) objective, Xcite 120 LED (Lumen Dynamics) light source, and Image EMX2 CCD (Hamamatsu) camera. Filter sets (center wavelength/bandwidth): mCerulean/mTFP1 excitation, 445/10 nm; mCerulean/mTFP1 emission, 485/30 nm; YPet/mVenus ex, 514/10 nm; Ypet/mVenus em, 540/21 nm. The experiment was performed twice and n ≥ 10 cells were analyzed for each experiment. Images shown in Fig. 3 are representative.

### Image processing

Raw images were imported into NIH ImageJ (v1.42q) for all processing operations including cropping, contrast adjustment, and quantitative analysis^51^. For each channel, 20 dark frames and 20 bright field images were stacked, converted to 32 bits, and median-filtered (radius 1), and each stack was averaged. The flat-field average was divided by the mean intensity of its central nine pixels to generate a normalized flat-field image. For each sample image, a median filter (radius 1) was applied and the master dark frame was subtracted. The resulting image was then divided by the normalized, master flat-field image, and the mean value of the detector offset was added back to the image. For ratiometric images and measurements, a binary mask was created by first averaging a series of Ypet images and then applying a threshold to highlight only regions exhibiting signal. The mask was applied to background-subtracted the FRET images were then divided by the donor image. Intensity-modulated ratiometric displays were generated using the Fire lookup table in ImageJ and a color lookup table was applied.
